# Intrinsic flexibility of porous materials; theory, modelling and the flexibility window of the EMT zeolite framework

**DOI:** 10.1107/S2052520615018739

**Published:** 2015-12-01

**Authors:** Rachel E. Fletcher, Stephen A. Wells, Ka Ming Leung, Peter P. Edwards, Asel Sartbaeva

**Affiliations:** a Department of Chemistry, University of Bath, Bath BA2 7AY, England; b Department of Chemistry, University of Oxford, South Parks Road, Oxford OX1 3QR, England; c Department of Chemistry, KOPRC, University of Oxford, South Parks Road, Oxford OX1 3QR, England

**Keywords:** framework, flexibility, geometric simulation, zeolite

## Abstract

Framework materials have structures containing strongly bonded polyhedral groups of atoms connected through their vertices. Typically the energy cost for variations of the inter-polyhedral geometry is much less than the cost of distortions of the polyhedra themselves – as in the case of silicates, where the geometry of the SiO_4_ tetrahedral group is much more strongly constrained than the Si—O—Si bridging angle. As a result, framework materials frequently display intrinsic flexibility, and their dynamic and static properties are strongly influenced by low-energy collective motions of the polyhedra. Insight into these motions can be obtained in reciprocal space through the ‘rigid unit mode’ (RUM) model, and in real-space through template-based geometric simulations. We briefly review the framework flexibility phenomena in energy-relevant materials, including ionic conductors, perovskites and zeolites. In particular we examine the ‘flexibility window’ phenomenon in zeolites and present novel results on the flexibility window of the EMT framework, which shed light on the role of structure-directing agents. Our key finding is that the crown ether, despite its steric bulk, does not limit the geometric flexibility of the framework.

## Abstract 2

Framework materials possess intrinsic flexibility which can be investigated using geometric simulation. We review framework flexibility properties in energy materials and present novel results on the flexibility window of the EMT zeolite framework containing 18-crown-6 ether as a structure directing agent (SDA).

## 1. Flexibility in framework materials and the flexibility window in zeolites   

Many mineral and material structures can be described as frameworks. They are made up of identifiable, strongly bonded polyhedral units connected together through vertices, edges or faces. The volume not contained within the polyhedra may be void space or represent ‘extra-framework’ sites where cations or small molecules can reside. Framework structures frequently contain tetrahedra, such as the archetypal SiO_4_ unit in framework silicates; octahedra, as in perovskites; or a mixture of both, as in garnets and spinels. Among synthetic materials, the metal-organic frameworks (MOFs) frequently display polyhedral coordination around metal centres with organic molecular bonding in the bridging ligands. Framework structures are of growing significance in energy applications as catalysts, ionic conductors or cathode materials for battery applications, and as storage materials for fuels or for carbon dioxide.

A particularly significant issue for framework structures is the phenomenon of intrinsic flexibility. When the geometry within the units making up the framework is significantly more strongly constrained than the geometry of the links between them, the properties of the structure can be strongly influenced by low-frequency collective modes of motion. Here the polyhedra in the framework rotate so that substantial amplitudes of atomic motion are achieved, but the energy cost is low as the strongest bonding constraints are not violated. Such collective modes may be observed directly in crystallography when they provide the ‘soft mode’ for a displacive phase transition, as in the quartz alpha/beta transition or the octahedral-tilting transformations of perovskites. Rotational motions can also contribute a large component of the thermal dynamic disorder in frameworks (Wells *et al.*, 2002 ▸, 2004 ▸), and can provide a mechanism for localized structural adaptation and defect accommodation. Framework flexibility can thus be very significant in accounting for the difference between local structure and the long-range crystallographic average.

We will briefly review some theoretical and simulation approaches for the investigation of framework flexibility, in particular the method of geometric simulation, and the ‘flexibility window’ phenomenon in zeolites. We provide some recent research results on the flexibility window of the EMT zeolite framework, including consideration of the presence of 18-crown-6 ether as an organic structure directing agent (oSDA). Our study sheds light on the subtle role of oSDAs in zeolite structure formation.

### 1.1. Simulations, rigid unit modes and geometric simulation   

A range of methods can be, and have been, used to model the behaviour of frameworks such as zeolites. Molecular dynamics (MD) in particular have proven to be a useful tool in obtaining diffusion coefficients for a small molecule mass transport through zeolite pores during catalysis (Fritzsche *et al.*, 2000 ▸), while density functional theory (DFT) can be a useful tool in modelling proposed reaction mechanisms occurring at the zeolite active sites. However, it can be both conceptually and practically useful to examine framework flexibility using specialized methods implementing a simplified physical model of the system. Such approaches are productive of insight and provide a flexibility-centred explanatory model within which experimental data on the one hand, and the results of simulations at higher levels of theory on the other, can be understood.

Flexibility can be investigated in reciprocal space using the ‘rigid unit mode’ (RUM) model (Giddy *et al.*, 1993 ▸; Hammonds *et al.*, 1994 ▸), a form of normal-mode analysis in which the interacting objects are the polyhedra making up the structure. Harmonic constraints are applied to connect the vertices of the polyhedra, which are coincident by construction in the input structure. These constraints penalize any separation of the connected vertices of two adjacent polyhedra, which can be thought of as a hypothetical ‘splitting’ of the bridging vertex atom, hence the term ‘split-atom model’. RUMs, in which the polyhedra move rigidly without distortion, appear as modes of zero frequency. Modes identified by the RUM model can be significant as soft modes for phase transitions and as contributors to negative thermal expansion (NTE) or negative Poisson ratio (auxetic) behaviour (Giddy *et al.*, 1993 ▸; Rimmer *et al.*, 2014 ▸). The *CRUSH* software implementing the RUM model, developed by Giddy, Dove and Hammonds, is available for download (see: http://www.ccp14.ac.uk/ccp/web-mirrors/crush/mineral_sciences/crush/).

The template-based geometric simulation approach (Wells *et al.*, 2002 ▸; Sartbaeva *et al.*, 2006 ▸; Wells & Sartbaeva, 2012 ▸, 2015 ▸) is a real-space simulation approach which includes both the explicitly present atoms, and a set of polyhedral template objects representing the bonding geometry of groups of atoms. Harmonic constraints link atoms to template vertices. The templates now need not match the input structure exactly by construction; rather, they can be defined as geometrically regular tetrahedra, octahedra *etc.* with an appropriate centre–vertex bond length. This approach offers a method to analyse a structure and quantify any distortions from perfect polyhedral geometry; it also offers a simulation approach of ‘geometric relaxation’, in which the positions of the atoms and templates are mutually relaxed so as to minimize the atom-template mismatches and also any steric overlap of nonbonded atoms. This approach and its implementation in the Geometric Analysis of Structural Polyhedra (*GASP*) software has recently been reviewed at length by two of the present authors (SAW and AS; Wells & Sartbaeva, 2015 ▸) and so we will not discuss it in great methodological detail here; some technical details can be found in §2. *GASP* software may be obtained from the authors by request: please email s.a.wells@bath.ac.uk.

### 1.2. Framework flexibility and material properties   

There are a number of systems in which framework flexibility has been identified as a key factor accounting for unusual material properties. For example, the motion of Li^+^ ions through a quartz structure (Sartbaeva, Wells & Redfern, 2004 ▸; Sartbaeva, Redfern & Lee, 2004 ▸; Sartbaeva *et al.*, 2005 ▸) is heavily influenced by collective flexible motions of the polyhedra (Wells *et al.*, 2004 ▸), leading to a very pronounced enhancement of conductivity (Hedvall effect) in the vicinity of the alpha/beta phase transition. Flexibility likewise affects the accommodation of a typical substitutional defect in silica: substitution of Al for Si, with the introduction of a nearby extraframework cation for charge balance (Goodwin *et al.*, 2006 ▸). On the introduction of the defect, changes in atomic positions propagate to large distances with amplitudes dropping off slowly with distance. However, the actual distortions of tetrahedral units in the framework drop away much more rapidly, being confined almost entirely to the nearest and next-nearest neighbour polyhedra. This ‘strain screening’ effect implies that defects in framework materials are essentially accommodated locally.

It is important to note that framework flexibility is not limited to the tetrahedral frameworks that we have discussed thus far. Similar effects are seen in, for example, perovskites, where the polyhedral units are octahedra, and octahedral tilting modes are important mechanisms of displacive phase transitions (Carpenter *et al.*, 2006 ▸). The addition of a rod-like molecule between two octahedral vertices in metal cyanide structures provides dramatically enhanced flexibility, leading to negative thermal expansion and auxetic behaviour (Goodwin *et al.*, 2004 ▸; Goodwin, Keen* et al.*, 2008 ▸; Conterio *et al.*, 2008 ▸; Goodwin, Calleja *et al.*, 2008 ▸).

Metal–organic frameworks (MOFs) can display a fascinating variety of flexibility properties (Sarkisov *et al.*, 2014 ▸; Rimmer *et al.*, 2014 ▸) depending on the topology of the framework, the intrinsic flexibility if any of the organic linkers, and the character of the bonding around the metal centre. The *GASP* software has recently been extended (Wells & Sartbaeva, 2015 ▸) to make it capable of handling the organic linkers in MOFs as well as the polyhedral coordination of metals.

Framework materials including spinels, phosphates and fluorosulfates are likely to be of increasing importance in the energy economy as battery materials in the next generation of lithium (and sodium) rechargeable batteries (Islam & Fisher, 2014 ▸; Fisher *et al.*, 2010 ▸; Eames *et al.*, 2015 ▸). There is also much current interest, for energy applications, in hybrid perovskites for solar cells (Leguy *et al.*, 2015 ▸; Walsh, 2015 ▸; Frost, Butler & Walsh, 2014 ▸; Frost, Butler, Brivio *et al.*, 2014 ▸; Brivio *et al.*, 2013 ▸) and in MOFs for gas storage. Properly taking account of framework flexibility will be critical in correctly modelling their properties for materials selection and device design. This highlights the need for simulations with sufficiently large system sizes to capture flexibility effects; multi-scale simulations and approaches such as geometric simulation will be needed.

### 1.3. The flexibility window in zeolites   

Zeolites are microporous crystalline aluminosilicates, whose unique, open three-dimensional framework structures are formed as networks of corner-linked tetrahedra (Baerlocher *et al.*, 2001 ▸). The structural framework chemistries of zeolite materials give rise to a range of characteristic chemical properties, widely exploitable in a multitude of industrial and domestic processes. Zeolites are most prominently used in the petrochemical industry as catalysts for cracking, alkylation and isomerization (Marcilly, 2003 ▸; Degnan, 2003 ▸). **EMT**-type zeolite, EMC-2, for example is an extremely effective industrial catalyst for the alkylation of isobutane with 2-butene (Stocker *et al.*, 1996 ▸; Rosenbach & Mota, 2005 ▸). A major ambition for the field is the development of ‘designer’ zeolites with tailored geometry for new catalytic applications, by the appropriate choice of synthesis conditions and organic structure-directing agents (oSDAs) whose shape is intended to template the specific pore geometry of the desired framework (Dhainaut *et al.*, 2013 ▸). Efforts to predict hypothetical tetrahedral frameworks as candidate structures have led to an embarrassment of riches; for example, the symmetry constrained inter-site bonding search (SCIBS) and energy minimization approach of Treacy and Foster has generated a database that now exceeds 5 million types (Foster *et al.*, 2005 ▸; Treacy *et al.*, 2004 ▸). The rate of synthesis of new zeolite structures experimentally, however, remains slow, thanks to two significant bottlenecks. Firstly, many of the predicted hypothetical structures are not in fact feasible, so identifying the truly feasible candidates is challenging. Secondly, to proceed from a candidate structure to the selection or design of an oSDA to template its formation is not a solved problem.

The application of template-based geometric simulation to zeolites using *GASP* has revealed an inherent zeolite geometric property: the ‘flexibility window’ (Sartbaeva *et al.*, 2006 ▸; Wells & Sartbaeva, 2012 ▸; Leung *et al.*, 2015 ▸; Kapko *et al.*, 2010 ▸; Dawson *et al.*, 2012 ▸). The window is the range of densities (more exactly, the range of unit-cell parameters) within which the tetrahedra of the zeolite framework can in principle retain their shape undistorted. Within the window the structure adapts through variation in the *T*–O–*T* linkages, which are considerably more flexible than their rigid O–*T*–O counterparts, and may feasibly exist over a range of angles (Wragg *et al.*, 2008 ▸; Baur, 1980 ▸). Existing zeolite frameworks, both natural and synthetic, characteristically possess a flexibility window, whereas the property is much rarer among hypothetical tetrahedral frameworks. This suggests that the existence of a flexibility window may be necessary for a structure to be accessible by hydrothermal synthesis. Zeolites are typically found under ambient conditions at densities corresponding to the more expanded edge of the window, and thus represent a form of ‘expanded condensed matter’. Further investigations have shown that the geometric property of the flexibility window is linked to the physics of zeolite framework behaviour, especially under pressure (Wells *et al.*, 2002 ▸; Sartbaeva *et al.*, 2008 ▸, 2012 ▸; Gatta *et al.*, 2009 ▸; Wells *et al.*, 2011 ▸).

A recent study of the flexibility window of the **FAU** framework in the presence of extra-framework water and methanol (Wells *et al.*, 2015 ▸) distinguished the *intrinsic* flexibility window of the empty framework from the *extrinsic* window limited by the presence of extraframework content. Since the geometric simulation model neglects long-range interactions, it is purely the steric effect of non-framework atoms that is considered. Even within this simple model, however, unexpected behaviour is observed. The presence of a combination of water and methanol within the β-cages of the **FAU** framework decreases the range of the flexibility window not only in compression but also in extension. When the cage contents are bulky and irregular in shape, cages may not be able to attain the geometries corresponding to the maximally expanded state of the empty framework.

## 2. Flexibility window in EMT zeolite framework   

**Figure 1:**
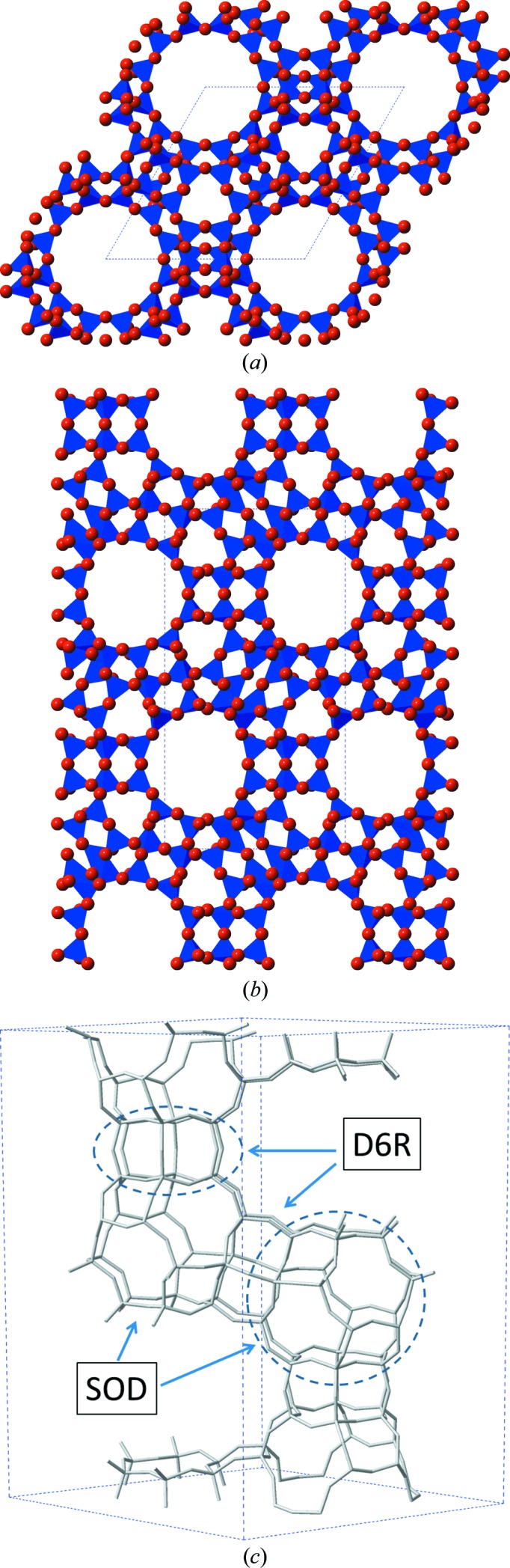
(*a*) **EMT** framework in polyhedral view along the *c* axis; (*b*) **EMT** framework in polyhedral view along the *a* axis; (*c*) **EMT** framework viewed as a network of T sites, with the *sod* and *d6r* building units highlighted.

Following the recent study of the flexibility window in cubic faujasite, this study focuses on the closely related hexagonal polymorph, EMC-2 (**EMT**-type zeolite; Delprato *et al.*, 1990 ▸). Like **FAU**, the **EMT**-type framework is composed of two secondary building units (SBUs), β-cages and double six-membered rings (D6Rs), which come together in a hexagonal array to form a two-dimensional periodic building unit (PerBU), the faujasite layer. Each faujasite layer contains a 12-ring window, and it is the variation in stacking between the two zeolites that affords two distinct framework types with different symmetry (Burkett & Davis, 1993 ▸; Baerlocher *et al.*, 1994 ▸). The ABA stacking of faujasite sheets affords the hexagonal **EMT**-type framework, with pairs of β-cages related to each other through a mirror plane. The cubic **FAU** framework arises from an ABC stacking sequence, each faujasite layer is rotated 60° to the one before it and each β-cage related to its partner by an inversion centre. The relative orientations of 12-ring windows give rise to ‘supercages’, characteristic of this framework polytype. The unit cells of both the hexagonal and cubic ‘faujasite’ possess four of these supercages. The cubic **FAU** framework contains four 12^4^ supercages, referred to as the *t-fau* cavity, whereas **EMT** contains two different supercages; one smaller 12^3^ supercage termed the *t-wof* cavity, and one larger 12^5^, known as the *t-wou* (Baerlocher *et al.*, 1994 ▸). The **EMT** framework is shown in Fig. 1 ▸.

During hydrothermal synthesis, the templating of these characteristic cavities by crown ether molecules with associated sodium ions can control the formation of the cubic or hexagonal form. In order to synthesize the hexagonal polymorph, not spontaneously formed in nature, 18-crown-6 is incorporated into the reaction mixture. The ether molecule forms a cation/crown complex with the sodium present in the initial synthesis gel. The sodium cation sits in the centre of the ether ring, and is held there through supramolecular cation–dipole interactions. The smaller 15-crown-5 affords the cubic polymorph, **FAU**. Recent studies have concluded that the generation of the smaller *t-wof* cavity, present only in the hexagonal polymorph, governs the resulting morphology of the final zeolite framework (Feijen *et al.*, 1994 ▸; Burkett & Davis, 1993 ▸). That is, the ring structure of the 18-crown-6/Na complex matches the geometry of one side of the *t-wof* cavity and leads to development of the **EMT** framework; the axis of the ether ring lies along the *c* axis of the hexagonal structure. A mixture of 15-crown-5 and 18-crown-6 ethers can lead to ordered intergrowths of the hexagonal and cubic polymorphs (Terasaki *et al.*, 1993 ▸). This study focuses on the flexibility window of the hexagonal **EMT** framework in both its calcined (empty) and crown-ether containing (as-synthesized) forms. Our specific objective is to determine whether the crown ether oSDA controls an extrinsic flexibility window in **EMT**.

### 2.1. Preparation of EMT structure for geometric simulations   

The all-atom input structure comprises a single EMC-2 (**EMT**) unit cell in *P*1 symmetry, using the coordinates obtained by Baerlocher *et al.* (1994 ▸) through Rietveld refinement. Each unit cell contains 96 tetrahedral units and the hexagonal unit-cell parameters of the ambient structure are *a*, *b* = 17.37, *c* = 28.36 Å. The framework was modelled in *GASP* as a purely siliceous framework; however, a Si—O bond length of 1.63 Å was assigned to reflect the presence of a small proportion of aluminium in the structure. Our results are not highly sensitive to the bond length: use of a shorter bond length of 1.61 Å, for pure silica, in this case slightly contracts the window but does not change its shape or character. The steric radius of oxygen in the framework, a key controlling parameter in the intrinsic flexibility window, was set at 1.35 Å as is conventional.

The as-synthesized crystal structure has well resolved 18-crown-6 ether molecules in the smaller *t-wof* cavities; in the crystallographic average structure, each cavity contains a superposition of two such molecules with 50% occupancy, as there is room in the cavity for only one molecule but its orientation – ‘facing up’ or ‘facing down’ – is random. For our input structure, a single copy of the ether was retained in each of the two *t-wof* cavities in the unit cell, one in each of the two possible orientations. The bond lengths and angles in the crown ether are taken from the input structure. Bonds are assigned in *GASP* between the central Na ion and its three coordinating O atoms to maintain the geometry of the complex. Since the H atoms in the CH_2_ groups of the ether are not resolved, a suitable effective radius must be assigned to the carbon atoms. We assign this radius by considering the closest approach between an ether C and a framework O atom in the crystal structure; this distance is 2.948 Å. We therefore assign a radius of 1.6 Å to the ether C atoms, so that the ether is just in contact with the framework initially. Rotation is permitted around all C—C and C—O bonds in the molecule, and the ether is not tethered to the framework so it is able to move and flex in response to changes in the cage geometry.

The presence of crown ether is detectable in the larger *t-wou* cages. However, these molecules are partially disordered and only some of the heavy atoms are resolved, indicating both positional and orientational disorder. Given this disorder and the larger size of the cavity, we did not attempt to model crown ether molecules in the *t-wou* cages, but rather neglected the disordered molecules and all other water and sodium ions. Our study therefore makes use of two input structures, one with crown ether molecules present in the *t-wof* cavities, and one without any extraframework content whatsoever, that is, an empty framework.

### 2.2. Flexibility window in EMT: results   

**Figure 2:**
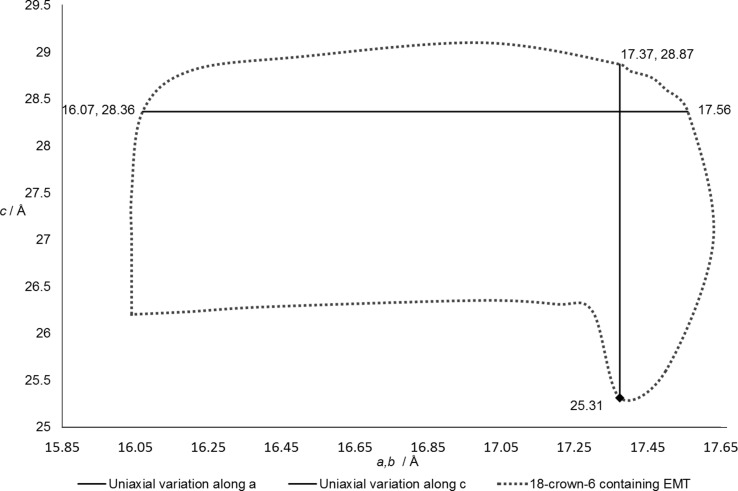
Extent of the flexibility window for the **EMT** framework during variation of the *a* and *c* parameters.

Since **EMT** is a hexagonal structure, its flexibility window is defined by variations of the two independent cell parameters, *a* (= *b*) and *c*. An initial investigation of the intrinsic flexibility window in **EMT** commences with the exploration of uniaxial variation of the parameters. We explore to an accuracy of 0.01 Å in each parameter. The *a* parameter can be expanded slightly to 17.56 Å, corresponding to a 2.20% increase in unit-cell volume, before the onset of extension in the Si—O bonds. In compression the parameter can be reduced substantially, to 16.07 Å, reducing the initial ambient unit-cell volume by 15.92%. This is consistent with previous reports of expanded-condensed matter behaviour in zeolite frameworks (Wells & Sartbaeva, 2015 ▸). For uniaxial variation along the *c* parameter, we saw a similar trend. The parameter can be extended to 28.87 Å, equivalent to a 1.80% expansion in unit-cell volume, compared with a 10.75% decrease in volume on compression to 25.31 Å. These limits, represented as solid, linear lines in Fig. 2 ▸, describe the uniaxial confines of the intrinsic flexibility window for empty EMC-2 (**EMT**).

Having established the preliminary limits to **EMT** flexibility, our next step was to determine points lying along the perimeter of the flexibility window. The results are depicted in Fig. 2 ▸ as dotted lines. The window has an almost rectangular shape in the *ac* plane, showing that there are substantial areas of phase space within which the parameters can be independently varied. In detail, however, the window has a strikingly nontrivial, ‘speech-bubble’ shape, showing that at the limits of compression there are complex interactions between the mechanisms of compression along different directions.

**Figure 3:**
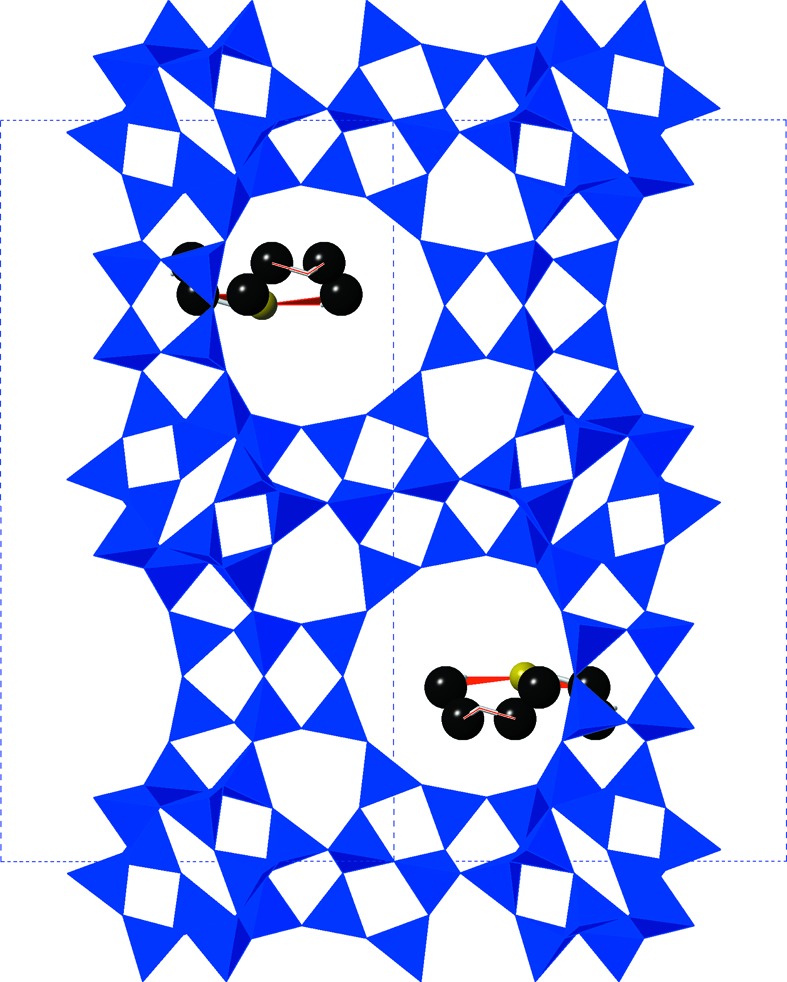
**EMT** framework under ambient conditions showing the location of well resolved crown ether molecules in the *t-wof* cages.

**Figure 4:**
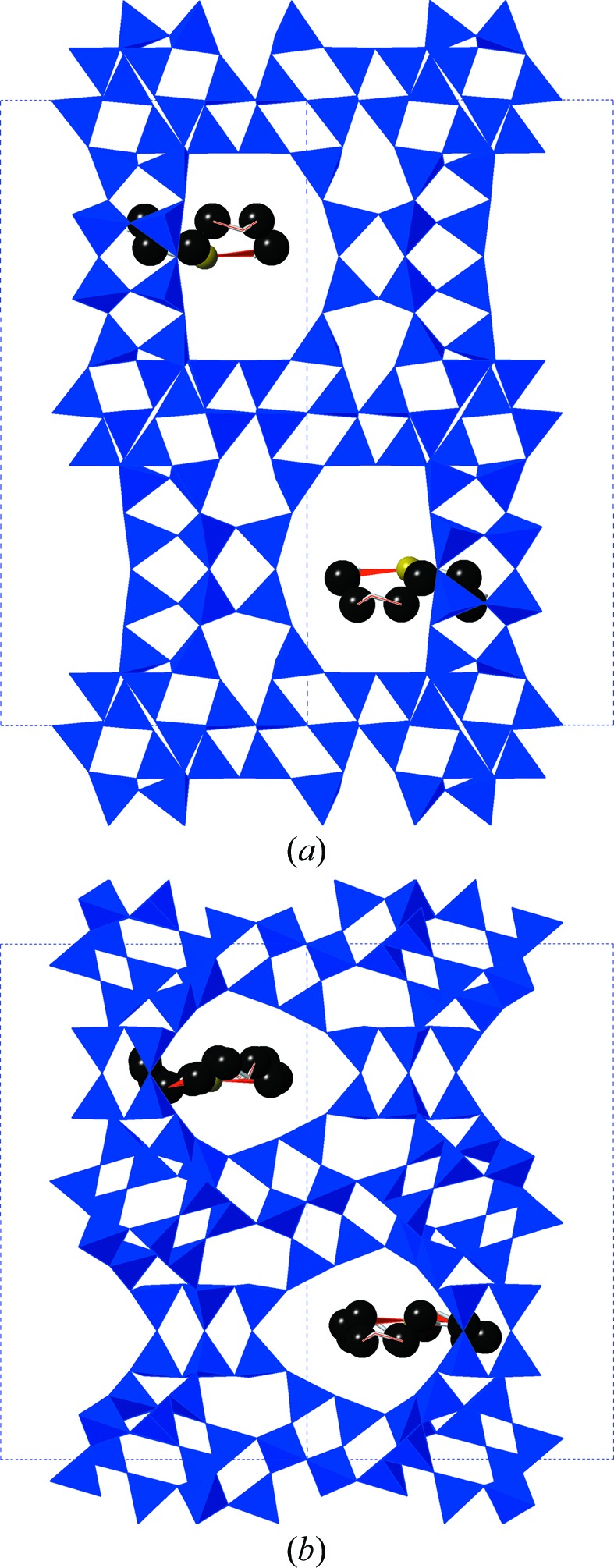
(*a*) **EMT** framework at the limit of geometric compression of the *a* parameter; (*b*) **EMT** framework at the limit of geometric compression of the *c* parameter.

Our next step was to carry out the same exploration of the flexibility window with Na^+^/crown ether complexes present in the *t-wof* cages of the **EMT** type framework. The crown ether is a bulky molecule which effectively fills one side of the *t-wof* cage, with the sodium ion and its coordinating ether O atoms lying in the mid-plane of the cage. The structure with crown ethers present is shown in Fig. 3 ▸. As noted, our ether carbon radius was chosen so that the ether is already in steric contact with the framework in the input structure. We therefore anticipated a substantial contraction of the flexibility window on inclusion of the ether oSDA in the simulation. The result we in fact obtain is entirely different: the **EMT** structure with ethers included displays an identical flexibility window to the empty framework. The window illustrated in Fig. 2 ▸ thus also applies to the framework with ethers present. The ether molecule has sufficient geometric flexibility that it can adapt to the contraction of the *t-wof* cage during the simulations, and the flexibility window remains under the control of intrinsic framework factors. A striking example of this adaptation is illustrated in Fig. 4 ▸, which shows the structure at the limit of compression of the *c* and *a* parameters. The cage itself displays substantial compression, and the ether molecule is in steric contact with the surrounding framework, yet the molecule can adapt to its surroundings so that it does not limit the contraction of the framework, and the degree of steric overlap remains of the order 0.01 Å or less.

## 3. Conclusions   

Intrinsic flexibility is a key feature of framework materials, and must be taken into account when seeking to understand their properties and behaviour. Energy materials including ionic conduction materials and battery cathode materials, zeolites, perovskites and MOFs fall into this class. Rigid unit mode and geometric simulation approaches can be valuable complements to conventional MD and DFT approaches in the investigation of such materials.

The unexpected result we have obtained in our study of **EMT** – that the crown ether oSDA does not geometrically limit the flexibility of the framework that it templates – highlights the subtle and complex role of SDAs in the zeolite synthesis process. Even in this case, with the striking match between the template and cage shape, the templating of the *t-wof* cavity clearly does not derive from a simple steric mechanism, with the ether defining a fixed shape onto which the framework assembles. Rather, the SDA must subtly influence the free-energy landscape to favour the formation and growth of this specific zeolite framework among the many possible metastable states of silica.
